# Molecular characterization of Orf virus isolates from Kodai hills, Tamil Nadu, India

**DOI:** 10.14202/vetworld.2019.1022-1027

**Published:** 2019-07-12

**Authors:** G. Nagarajan, R. Pourouchottamane, G. B. Manjunatha Reddy, R. Yogisharadhya, K. Sumana, S. Rajapandi, G. Murali, S. M. K. Thirumaran, P. K. Mallick, A. S. Rajendiran

**Affiliations:** 1 Southern Regional Research Centre, ICAR-Central Sheep and Wool Research Institute, Kodaikanal, Tamil Nadu, India; 2 ICAR-Central Institute for Research on Goats, Makhdoom, Uttar Pradesh, India; 3 ICAR-National Institute of Veterinary Epidemiology and Disease Informatics, Yelahanka, Bengaluru, India; 4 ICAR-Central Sheep and Wool Research Institute, Avikanagar, Rajasthan, India

**Keywords:** B2L gene, Kodai hills, Orf virus, phylogenetic analysis, sheep

## Abstract

**Aim:**

The present study was carried out to find out the causative agent of exanthematous skin lesions in sheep maintained by Southern Regional Research Centre, Mannavanur, Kodai hills, Tamil Nadu.

**Materials and Methods:**

Polymerase chain reaction (PCR) with Orf virus (ORFV) B2L gene-specific primers was carried out by employing the total genomic DNA isolated from the scabs as the template. The ORFV isolates from Kodai hills were characterized by the use of bioinformatics tools.

**Results:**

The amino acid identity of ORFV isolate 1 from Kodai hills is having 98.14%, 96.29%, and 83.59% identity with reference strains of ORFV, *Pseudocowpox* virus, and bovine papular stomatitis virus, respectively. Phylogenetic analysis revealed that ORFV isolates from Kodai hills clustered with the other ORFV isolates from different geographical areas of India.

**Conclusion:**

The etiological agent of exanthematous skin lesion among sheep of Kodai hills is ORFV.

## Introduction

Orf, popularly called as contagious ecthyma, is considered as an acute, contagious, debilitating, and economically important zoonotic viral skin disease in sheep and goats. The etiological agent is Orf virus (ORFV) belonging to the genus *Parapoxvirus* (PPV), within the subfamily *Chordopoxvirinae*, of the family *Poxviridae* [1]. When compared to sheep, this disease is more severe in goats. In addition to small ruminants, this virus infects alpacas, camels, reindeer, deer, pronghorn antelope, wapiti, and seal squirrels [2].

As in the case of other poxvirus infections, contagious ecthyma is also appreciated with the onset of papules, vesicles, pustules, and followed by unique scabs and the said lesions are restricted to the facial region in most of the cases [3].

Orf viral infection is pandemic in distribution and the remarkable outbreaks have been noticed during late summer, fall, and winter on pasture and in feedlots. This epitheliotropic virus is hardy in nature and could remain viable for months or even years in conducive environment, and the transmission of this virus commonly happens through contact from infected to susceptible animals [2]. In spite of the easy diagnosis of Orf disease based on the clinical symptoms, final confirmation was done using the molecular diagnostic approach of polymerase chain reaction (PCR) studies [4,5].

Molecular technique, namely, PCR based on B2L gene-specific primers of ORFV was extensively used for the confirmatory diagnosis of contagious ecthyma in sheep and goats at Mukteswar, India [6], sheep herd in Northeast China [7], sheep and goats of Kashmir Himalayas [8], Black Bengal goats from Tripura state, India [9], and small ruminants in Northwest Ethiopia [5]. Adedeji *et al*. [10] carried out the diagnosis of Orf viral outbreak among 60 male West African dwarf goats in an experimental farm in Uyo, Akwa Ibom State, Nigeria, by PCR technique.

Kodaikanal Wildlife Sanctuary, located at Kodai hills, Tamil Nadu, in Southern part of Indian subcontinent, is the habitat for many of the endangered fauna such as elephants, tiger, panther, bison, and deer species such as Sambar, Chital, Barking deer, Nilgiri Tahr and so on. Kodaikanal Wildlife Sanctuary also supports around 44 mega mammalian fauna.

According to the International Zonal Classification, the climate of Kodaikanal broad falls under “36^th^ Medium Tropical Transitional Bioclimate.” Relative humidity is high during retreating Northeast monsoon season (October-mid-December). The mean temperature of Kodaikanal taluk is 15.93°C with a mean summer temperature of 17.29°C (June, July, and August) and mean winter (December, January, and February) temperature of 14.10°C. The average annual rainfall is 1436.87 mm. The climate is quasi-temperate, despite low latitude, with moderate temperatures of low annual range.

The forests of Kodaikanal Wildlife Sanctuary fall between 77° 16’ and 77° 45’ of East longitude and 10° 20’ and 10° 5’ of North latitude. It is regarded as one of the important protected areas in the Western Ghats due to its ecological, floral, and geomorphological significance. The Western Ghats are recognized as one of the three mega centers for endemism in the country.

Southern Regional Research Centre (SRRC), one of the regional centers of ICAR-Central Sheep and Wool Research Institute (ICAR-CSWRI), is located within the Kodai hills and is 32 km away from Kodaikanal town. SRRC is well known for the maintenance of exotic breeds of sheep (Bharat Merino and Avikalin) as well as broiler breeds of rabbits (White Giant and Soviet Chinchilla) as well as for its services in catering the needs of the farmers from southern part of the country.

Unexpectedly and 1^st^ time in the history of its existence, both lambs and adult sheep being maintained by SRRC were exhibiting exanthematous skin lesions around the facial region during August 2015. No animal was having the problems of limping.

Further, all the animals at SRRC were regularly vaccinated against foot-and-mouth disease. In addition, there is no report about the prevalence of vector for Bluetongue in Kodai hills. Therefore, based on the first author’s expertise in contagious ecthyma among camels and restriction of the lesions only to the facial region of the affected animals, the outbreak was caused by ORFV in the affected sheep.

The importance of molecular characterization of ORFV in an outbreak from a new area would be useful to find out the mutation (if any) within the gene sequences it possesses and eventually it is useful in the formulation of the suitable preventive and control measures against the virus isolate from that particular geographical area. Further, B2L gene-based PCR technique is the ideal one for the confirmative diagnosis of the infections caused by PPVs.

The present study was carried out with the aim of detection of the causative agent of the disease in sheep by employing B2L gene-based PCR and characterized the same using the bioinformatics tools.

## Materials and Methods

### Ethical approval

All animal experiments were performed according to protocols approved by the Institutioanl Committee of Southern Regional Research Centre, ICAR-Central Sheep and Wool Research Institute for use and care of animals.

### Scab materials

During the time of outbreak, the total numbers of animals present were 461 and the number of animals affected was 85. There was no correlation between the time point and other parameters.

Scab materials and blood samples were collected from five severely affected lambs of <3 months of age (either sex of Bharat Merino) being maintained by SRRC (ICAR-CSWRI), Mannavanur, Kodaikanal, Tamil Nadu, India, and sent for the confirmatory diagnosis at ICAR-National Institute of Veterinary Epidemiology and Disease Informatics (NIVEDI), Bangalore, India.

### PCR

Skin scabs were collected from the suspected lesions of contagious ecthyma and stored at −20°C until use. DNA was extracted from the skin scabs using Genei Ultrapure^™^ Mammalian Genomic DNA Purification Kit – Tissues (Bangalore GeNei Pvt. Ltd., India) according to the manufacturer’s instructions. Reaction volumes for the PCR of 50 μl were used and contained 5 μl of 10× buffer with 15 mM MgCl_2_, 10 mM of each dNTPs, 100 pmol of each oligonucleotide primer, 100 ng of DNA sample, and 3U Taq DNA polymerase. The envelope protein (B2L) encoding gene of ORFV obtained from Kodai hills, India, was amplified from the genomic DNA isolated from the sheepskin scabs infected with contagious ecthyma by PCR using the primers designed using the sequence of the ORFVNZ2 strain (GenBank accession Number DQ184476). The sequences of the primers were OVB2LF1: TCC CTG AAG CCC TAT TAT TTT TGT G (25mer) and OVB2LR1: GCT TGC GGG CGT TCG GAC CTT C (22mer) [6].

The reaction mixture was subjected to initial denaturation of the template at 94°C for 5 min in a thermal cycler (Eppendorf, Germany). Cycling conditions for PCR were 35 cycles of 60 s at 94°C, 60 s at annealing temperature of 55°C, and 60 s at 72°C followed by a final extension for 10 min at 72°C. In the PCR, the total genomic DNA isolated from the goat infected with capripoxvirus and sheep infected with sheeppox was used as positive and negative controls, respectively.

### Sequencing of PCR amplified DNA fragments

The PCR amplified products from five individual scab materials were analyzed in 1.2% agarose gel. Out of five positive amplicons, only three amplicons were purified using the gel extraction kit. After the gel purification, sequencing of the PCR products in both directions was carried out at M/s. Xcelaris, Ahmedabad, India, using the gene-specific primers. Bioinformatic analysis of the sequences was carried out using CLC Genomics Workbench 8.5 (Qiagen). Nucleotide sequences corresponding to B2L gene of three ORFV isolates from Kodai hills were submitted to Genbank and accession number obtained (from KU597728 to KU597730). The determined nucleotide sequences and the deduced amino acid sequences of the B2L were analyzed with the Basic Local Alignment Search Tool (BLAST) program (NCBI) search of GenBank. The evolutionary history was inferred using the neighbor-joining method [11]. The bootstrap consensus tree inferred from 1000 replicates was taken to represent the evolutionary history of the taxa analyzed [12]. The evolutionary distances were computed using the Tamura 3-parameter method [13] and the evolutionary analyses were conducted in MEGA7.0 software (Pennsylvania University, USA) [14].

## Results and Discussion

Since the time of its genesis, the sheep maintained at SRRC had not faced an outbreak of Orf viral infection and the present study is the first of its kind in nature. Hence, the present study is quite surprising and interesting one.

**Figure-1:**
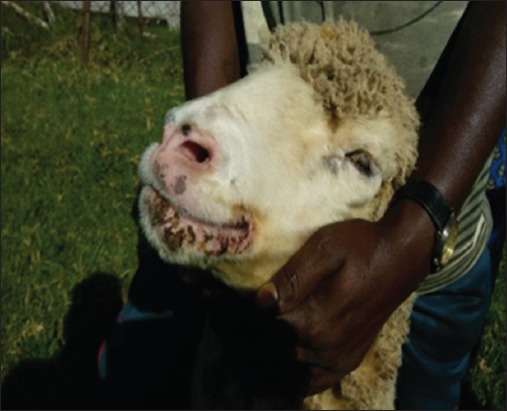
A Bharat Merino suckling lamb showing the exanthematous skin lesions.

In addition to all the suckling lambs, adults were also showing severe exanthematous skin lesions in the facial region (Figure-1). The morbidity was 100% with zero mortality. All the affected animals were given ad most care in terms of the usage of antibiotics and supportive therapy regimen to avoid death due to secondary bacterial infection and starvation. As stated in earlier reports [15], contagious ecthyma is seldom fatal and the infected animals normally recover within a month; but one can expect the mortality among the ailing animals if they are not attended in time to prevent secondary complications such as bacterial infections or myiasis.

Due to the richness in laboratory facilities and popularity in animal disease, diagnosis in Southern India, ICAR-NIVEDI, Bangalore, was chosen as the place for the molecular detection and subsequent characterization of the causative agent in the present outbreak among sheep.

There was an amplification of a fragment of around 1200 bp size observed in all the five DNA samples extracted from five different scab materials. In the present study, the length of the B2L gene of all the three ORFV isolated from sheep of Kodai hills is 1137 bp as expected. The sequence data obtained using gene-specific primers for all three PCR amplicons were identical and the consensus sequence was confirmed by the use of BLAST program.

**Figure-2:**
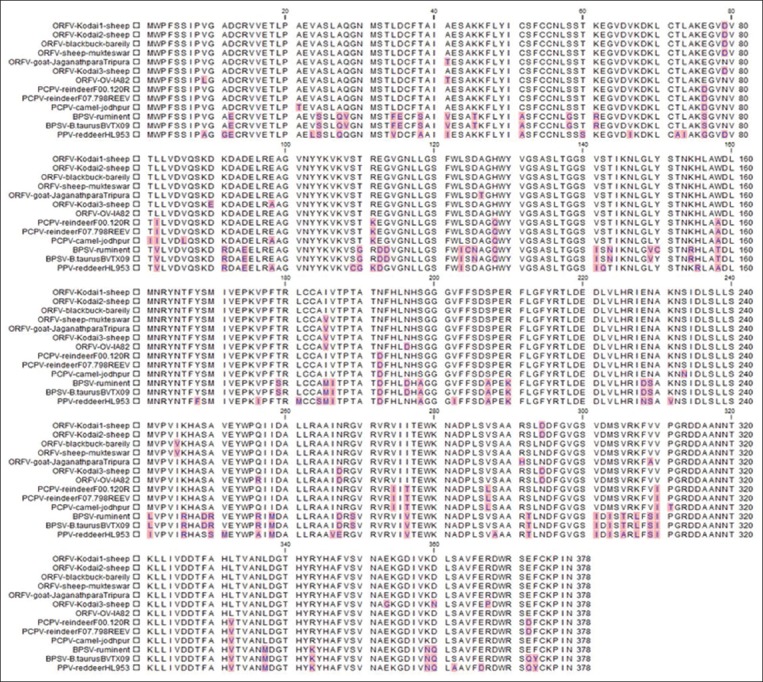
Alignment of amino acid sequences of B2L gene of Orf virus/Kodai with other *Parapoxvirus*.

The complete nucleotide sequences of B2L encoding gene of three sheep ORFV from Kodai hills, India, were compared to corresponding amino acid sequences from four ORFV from different geographical areas of India and six other PPV sequences available in the NCBI database (Figure-2). The open reading frame (ORF) of B2L encoding gene of sheep ORFV from Kodai hills is 378 amino acid encoding a polypeptide of 45.4 kDa.

As far as the sequence analysis concerned, B2L encoding gene of sheep ORFV isolate 1 from Kodai hills showed 97.62-99.20% and 98.14-100.00% sequence identity at the nucleotide and amino acid level, respectively, with other two ORFV isolates from Kodai hills. With the remaining four ORFV isolates from different geographical areas of India, ORFV isolate 1 from Kodai hills shared 97.36-97.97% and 98.14-99.47% identity at nucleotide and amino acid level, respectively. On the other hand, the amino acid identity of ORFV isolate from Kodai hills was having 98.14%, 96.29%, and 83.59% homology with reference strains of ORFV (GenBank accession number AY386263.1), *Pseudocowpox* virus (PCPV) (GenBank accession number GQ329669.1), and bovine papular stomatitis virus (BPSV) (GenBank accession No. AY424973.1), respectively (Table-1).

**Table 1:** Percent Nt and aa identity of B2L gene of ORFV/Kodai 1 isolate with other PPVs.

Virus isolate/Host	NCBI accession No	Nt(%)	aa(%)
ORFV-Kodai 1/Sheep	KU597728.1	-	-
ORFV-Kodai 2/Sheep	KU597729.1	99.2	100
ORFV-Kodai 3/Sheep	KU597730.1	97.62	98.14
ORFV-OV-IA82/Lamb	AY386263.1	97.97	98.14
ORFV/Bareilly/black buck/2013	KT191487.1	98.32	99.47
ORFV-Mukteswar/Sheep	GU139356.1	98.06	99.2
ORFV-Jaganathpara-Tripura/Goat	KT935588.1	97.36	98.14
PCPV-Jodhpur/Camel	GQ390365.1	93.22	94.97
PCPV-F00.120R/reindeer	GQ329669.1	93.57	96.29
PCPV-F07.798REEV/reindeer	JF773692.1	93.57	96.29
BPSV reference strain	AY424973.1	84.34	83.59
PPV-HL953/Red deer	KM502564.1	82.58	81.74
BPSV-BVTX09/*Bos taurus*	KM875472.1	84.16	83.59

ORFV=Orf virus, PPV=*Parapoxvirus*, PCPV=*Pseudocowpox* virus, BPSV=Bovine papular stomatitis virus, aa=Amino acid, Nt=Nucleotide

**Figure-3:**
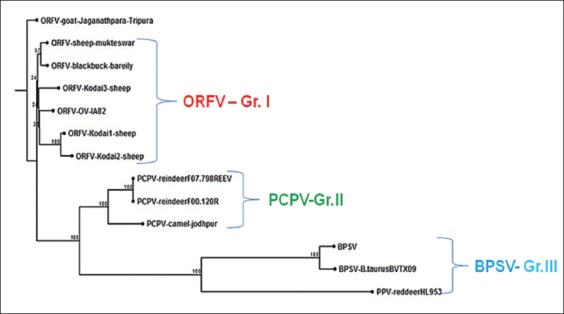
Phylogenetic tree based on nucleotide sequences of B2L gene of Orf virus (ORFV) isolates from Kodai hills, India, with ORFV isolates from different geographical areas of India and other *Parapoxvirus* from other animal species. The evolutionary history was inferred using the neighbor-joining method. The bootstrap consensus tree inferred from 1000 replicates was taken to represent the evolutionary history of the taxa analyzed. The evolutionary distances were computed using the Tamura 3-parameter method and the evolutionary analyses were conducted in MEGA7.

Analysis of the phylogenetic tree framed using amino acid sequences of the B2L encoding gene revealed that the three ORFV isolates from sheep inhabiting Kodai hills clustered with the other ORFV isolates from different geographical areas of the world (Group 1), published earlier, supported by high bootstrap values. PCPV isolates from reindeer and the camel clustered together forming another group (Group 2) while Group 3 is formed by BPSV reference strain, BPSV isolate of *Bos taurus* and PPV isolate from Red deer (Figure-3). In this study, ORFV isolates from sheep inhabiting Kodai hills, Tamil Nadu, India, were confirmed by PCR using B2L gene and sequenced for the 1^st^ time. No human case of infection was observed in the present outbreak. Similar to the earlier reports [4,5,7], PCR is the ideal test for the confirmatory diagnosis of Orf viral outbreak in the present study also.

Since Kodai hills are the habitat for wild ruminants such as bison and deer species including Sambar, Chital, Barking deer, and Nilgiri Tahr, a more detailed comparison of the PPVs from sheep with PPVs from other mammals at genetic level (if any) would be useful to find out the important factors responsible for the present ORFV outbreak among sheep in Kodai hills as well as to explain the host range of different PPVs and orthopoxviruses. Peralta *et al*. [4] proposed that the introduction of apparently asymptomatic animals into a new flock would be the starting point of the contagious ecthyma outbreaks and this would emphasize the essentiality of the knowledge pertaining to the health status of the livestock species sold among the farms for the purpose of the prevention of spread of the microbes across the world.

In addition to the stoppage of trade of animal and animal products across the national and global level, the outcome of contagious ecthyma would also result in production loss in livestock as well as reduction in the market value of various livestock products including meat, leather, and wool [2,16].

The sequence information obtained in the present study would be useful for the development of a suitable vaccine against ORFV trains circulating in Southern parts of India. Retrieval of nucleotide sequence data of ORFV published by various research groups from different geographical areas of the nation and nations from the public domains would enlighten the knowledge of the Orf experts, and it is further extremely useful in the development of ideal therapeutic and suitable control measures for its eradication [4].

## Conclusion

The exanthematous skin lesions in sheep maintained at Kodai hills, Tamil Nadu, India, were caused by ORFV. For the molecular epidemiological point of view, additional sequence analysis and functional assays of various immunomodulatory protein genes of the sheep ORFV need to be conducted for the purpose of developing the suitable control measures of PPV infections in Kodai hills in the future.

## Authors’ Contributions

GN reported the outbreak. GN, SR, and GM collected the scab materials from sheep. GN and GBMR designed the experiment. KS, RY, and GBMR carried out the PCR work and sequencing. RP, PKM, and SMKT were involved in analysis of the data as well as in scientific discussion. ASR monitored the overall work. All authors read and approved the final manuscript.
